# Supplementary material for: MetaRibo-Seq measures translation in microbiomes
Source: Nat Commun. 2020 Jun 29;11:3268. doi: 10.1038/s41467-020-17081-z (PMC7324362; doi:10.1038/s41467-020-17081-z)
Supplement: Supplementary file 10 — Supplementary Data 7 [file 41467_2020_17081_MOESM10_ESM.zip › File2/Confidence_High_Taxonomy/220609_out.krona.html]

Javascript must be enabled to view this page.

members
magnitude
magnitudeUnassigned
count
unassigned
taxon
rank

220609\_out

107

107
2
superkingdom

107
phylum
976

200643
107
class

107
1
order
171549

SRS140492\_contig\_number\_32888

171552
1
family

838
1
genus

1
species
1262937

SRS044535\_contig\_number\_23033

171550
3
family

3
239759
genus


SRS149784\_contig\_number\_22028
1
species
28117


SRS015794\_contig\_number\_8413
1
1872444
species


SRS011405\_contig\_number\_8864
species
1
1262694

88
family
815


SRS014235\_contig\_number\_contig-100\_370.227209SRS015369\_contig\_number\_6088SRS015782\_contig\_number\_47719SRS019397\_contig\_number\_9700SRS047433\_contig\_number\_19442SRS050925\_contig\_number\_24510SRS077194\_contig\_number\_32724
816
genus
7
88


SRS077849\_contig\_number\_10917SRS893342\_contig\_number\_11404SRS893378\_contig\_number\_21065
371601
3
species


SRS016056\_contig\_number\_5618SRS017247\_contig\_number\_728SRS024075\_contig\_number\_34SRS047014\_contig\_number\_35298SRS077552\_contig\_number\_11750SRS893358\_contig\_number\_12405
6
46506
species


SRS140645\_contig\_number\_567
1
species
28116


SRS021484\_contig\_number\_18174
species
1
1263037

45
species
47678

SRS011084\_contig\_number\_11715SRS012273\_contig\_number\_9404SRS013521\_contig\_number\_13681SRS015264\_contig\_number\_22833SRS016095\_contig\_number\_7316SRS016989\_contig\_number\_contig-100\_635.94447SRS017307\_contig\_number\_12755SRS017433\_contig\_number\_17418SRS017821\_contig\_number\_12255SRS018133\_contig\_number\_contig-100\_63.83163SRS018623\_contig\_number\_92SRS018836\_contig\_number\_25765SRS018888\_contig\_number\_6357SRS019910\_contig\_number\_24731SRS023526\_contig\_number\_26721SRS043667\_contig\_number\_249SRS043768\_contig\_number\_56SRS045826\_contig\_number\_22275SRS046068\_contig\_number\_945SRS047044\_contig\_number\_157SRS049995\_contig\_number\_36881SRS050299\_contig\_number\_36SRS052027\_contig\_number\_contig-100\_1402.112547SRS053398\_contig\_number\_24913SRS064757\_contig\_number\_25330SRS075878\_contig\_number\_18750SRS077024\_contig\_number\_3478SRS077086\_contig\_number\_2895SRS077454\_contig\_number\_24SRS078176\_contig\_number\_21737SRS078665\_contig\_number\_2465SRS1041037\_contig\_number\_10963SRS1041141\_contig\_number\_140SRS104327\_contig\_number\_1054SRS142890\_contig\_number\_24965SRS143070\_contig\_number\_14828SRS143523\_contig\_number\_24277SRS147039\_contig\_number\_294SRS147557\_contig\_number\_7622SRS147977\_contig\_number\_40SRS148091\_contig\_number\_14051SRS148196\_contig\_number\_41578SRS148253\_contig\_number\_28SRS149244\_contig\_number\_11977SRS893285\_contig\_number\_317

1
species
1896974

SRS143722\_contig\_number\_7679


SRS049402\_contig\_number\_15707SRS049896\_contig\_number\_935SRS147139\_contig\_number\_contig-100\_184.212826SRS893279\_contig\_number\_14864
4
species
818

species
2
817

SRS055966\_contig\_number\_220SRS077589\_contig\_number\_contig-100\_691.54099


SRS023346\_contig\_number\_7244SRS054905\_contig\_number\_21578SRS057717\_contig\_number\_2350SRS142923\_contig\_number\_923
585544
4
species

1
1739517
species

SRS064557\_contig\_number\_115

1
species
674529

SRS062427\_contig\_number\_73

1
246787
species

SRS016018\_contig\_number\_5374


SRS022071\_contig\_number\_contig-100\_459.225063
species
1
1907658


SRS014287\_contig\_number\_123SRS014923\_contig\_number\_27284SRS021153\_contig\_number\_182SRS024009\_contig\_number\_10599SRS045244\_contig\_number\_130SRS051882\_contig\_number\_9739SRS063127\_contig\_number\_3023SRS098827\_contig\_number\_contig-100\_10.73715SRS149879\_contig\_number\_4714
821
9
species

species
1
820

SRS1041033\_contig\_number\_19476

2005525
2
family

2
375288
genus


SRS144362\_contig\_number\_38380
species
1
823

1
46503
species

SRS143991\_contig\_number\_21781

family
6
1853231

3
4
genus
283168

SRS016335\_contig\_number\_29514SRS017521\_contig\_number\_48878SRS893383\_contig\_number\_34651

28118
1
species

SRS058770\_contig\_number\_16073

2
genus
574697

544645
1
species

SRS142599\_contig\_number\_contig-100\_549.188704


SRS103987\_contig\_number\_32822
species
1
544644

6
171551
family


SRS013940\_contig\_number\_17631SRS014459\_contig\_number\_38163SRS015065\_contig\_number\_36323SRS019685\_contig\_number\_4081SRS024132\_contig\_number\_23328SRS148424\_contig\_number\_contig-100\_177.168768
6
1952631
species
